# Mortality prediction of the nutrient profile of the Chilean front-of-pack warning labels: Results from the Seguimiento Universidad de Navarra prospective cohort study

**DOI:** 10.3389/fnut.2022.951738

**Published:** 2022-10-21

**Authors:** Vanessa Bullón-Vela, Carmen Sayón-Orea, Clara Gómez-Donoso, J. A. Martínez, Miguel A. Martínez-González, Maira Bes-Rastrollo

**Affiliations:** ^1^Department of Preventive Medicine and Public Health, School of Medicine, University of Navarra, Pamplona, Spain; ^2^Navarra Institute for Health Research, Pamplona, Spain; ^3^Biomedical Research Centre Network on Physiopathology of Obesity and Nutrition, Institute of Health Carlos III, Madrid, Spain; ^4^Global Obesity Centre, Institute for Health Transformation, Deakin University, Geelong, VIC, Australia; ^5^Department of Nutrition, Food Sciences and Physiology, School of Pharmacy and Nutrition, University of Navarra, Pamplona, Spain; ^6^Madrid Institute for Advanced Studies in Food, Madrid, Spain; ^7^Department of Nutrition, Harvard T.H. Chan School of Public Health, Boston, MA, United States

**Keywords:** all-cause mortality, cancer, CVD, front-of-pack nutrition labels, warning label, Nutri-Score

## Abstract

**Background and aims:**

Front-of-Pack (FoP) nutrition labelling has been established as a policy, empowering consumers to choose healthy food options for preventing diet-related non-communicable diseases. This study aimed to evaluate the association between the nutrient profile underlying the Chilean warning label score and all-cause mortality and to conduct a calibration with the Nutri-Score in a large cohort of Spanish university graduates.

**Materials and methods:**

This prospective cohort study analysed 20,666 participants (8,068 men and 12,598 women) with a mean (standard deviation) age of 38 years (±12.4) from the SUN cohort. Dietary food intake was assessed by a validated semi-quantitative food-frequency questionnaire at baseline and after 10 years of follow-up. The warning label score was calculated by considering the threshold of nutrients (sugar, saturated fat, and sodium) and energy density per 100 g/ml of product, as established by Chilean Legislation. Participants were classified according to quartiles of consumption of daily label score: Q1 (≤5.0), Q2 (>5.0–7.1), Q3 (>7.1–9.8), and Q4 (>9.8). Time-dependent, multivariable-adjusted Cox models were applied. To compare the performance of the warning label score and Nutri-Score to predict mortality, we used the Akaike information criterion (AIC) and Bayesian information criterion (BIC) methods.

**Results:**

During a median of 12.2 years of follow-up, 467 deaths were identified. A higher score in the warning label values (lower nutritional quality) was associated with an increased risk of all-cause mortality [HR (95% CI) Q4 vs. Q1: 1.51 (1.07–2.13); *p*-trend = 0.010] and cancer mortality [HR (95% CI) Q4 vs. Q1: 1.91 (1.18–3.10); *p*-trend = 0.006]. However, no statistically significant association was found for cardiovascular mortality. Furthermore, the warning label score and Nutri-Score exhibited comparable AIC and BIC values, showing similar power of prediction for mortality.

**Conclusion:**

A diet with a higher warning label score (>9.8 per day) was a good predictor of all cases and cancer mortality in a large Spanish cohort of university graduates. Also, the warning label score was capable to predict mortality as well as the Nutri-Score. Our findings support the validity of the warning label score as a FoP nutrition labelling policy since it can highlight less healthy food products.

## Introduction

According to the World Health Organization (WHO), non-communicable diseases (NCDs), including cardiovascular diseases (CVDs), cancer, diabetes, respiratory diseases, and others, are still the world’s leading cause with 71% of premature death between 30 and 69 years of age (1). NCDs share key modifiable behavioural risk factors related to health-related behaviours such as tobacco use, excessive alcohol consumption, and eating habits (1, 2). In particular, it has been estimated that a suboptimal diet is associated with 11 million deaths and 255 million disability-adjusted life-years (2). Among these dietary risk factors, the lower intake of fruits and whole grains stands out (2, 3). Industry process methods such as drying, pasteurization, freezing, and others are important to extend the life of foods. However, the manufactured formulation of ultra-processed foods (UPF) uses many ingredients and employs several processing methods, making the final product high- or ultra-palatable (4). Ingredients include sugar, salt, stabilizers, preservatives, and sources of energy such as oils, fats, hydrogenated fats, and fructose corn syrup, and other ingredients are cosmetic additives to emulate sensorial qualities of unprocessed or minimally processed food (4). Existing evidence suggests that UPF is closely related to poorer diet quality and increased risk of mortality (5, 6). In this context, improving the nutritional quality of food products represents a crucial strategy to reduce the NCDs burden.

Over the past years, some governments have implemented Front-of-Package (FoP) nutrition labels as a part of their strategy to mitigate the global burden of diet-related NCDs (7, 8). FoP labelling complements nutrient declaration, helping consumers to identify the healthiest or unhealthiest options, and prompting the food industry to reformulate their products (7). Many FoP nutrition label formats such as stars, traffic lights, spectrum rating (Nutri-Score), and stop sign warnings (warning label score) are currently used worldwide (7). The warning label was first adopted in Chile for packaged food and drinks with unhealthy levels of sugar, saturated fats, sodium, and/or calories (7, 9). Similar warning label systems have been adopted or are being considered in Peru, Uruguay, Argentina, Mexico, and Brazil (7, 10). Whereas in Europe, many countries have adopted the Nutri-Score, a five-colour FoP label based on nutritional criteria established by the Food Standard Agency Nutrient Profiling System (FSA-NPS) (11). The last few years have witnessed huge growth in the number of studies suggesting an association between the Nutri-Score nutrient profile and the increased risk of mortality and NCDs the effect of (6, 12–16). However, there has not been any research evaluating the effect of food consumption with Chilean warning label scores on health outcomes. To address of our study this gap in the research outlined earlier, the main objective of our study was to prospectively assess the association between the nutrient profile underlying the Chilean warning label score and mortality. Secondly, we investigated the prediction power for mortality by comparing the warning label score and Nutri-Score nutrition profiles.

## Materials and methods

### Study population: Seguimiento Universidad de Navarra cohort project

The Seguimiento Universidad de Navarra (SUN) project is an ongoing, multipurpose, prospective, and dynamic cohort study of Spanish university graduates (17). This cohort study investigates sociodemographic, lifestyle, and dietary factors related to the development of NCDs (17). The Institutional Review Board of the University of Navarra approved the study protocol. Individuals gave consent to participate in the study if they complete the first self-administrated questionnaire. All study procedures were conducted according to the Declaration of Helsinki. This study was registered at clinicaltrials.gov (NCT02669602).

**FIGURE 1:**
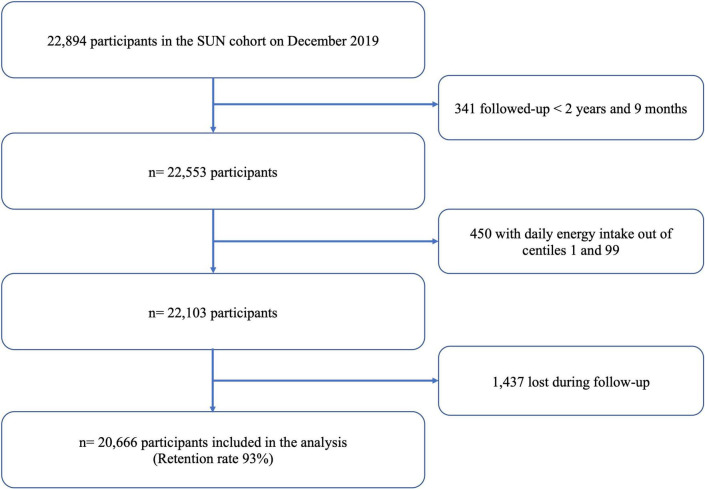
Flow chart for participants included in the SUN project.

Details of the design and methods of the SUN cohort have been previously published (17). In brief, the recruitment of volunteers started in December 1999 at the University of Navarra and other Spanish universities. Data collection and follow-up are done every 2 years by email or ordinary mail questionnaires. By December 2019, the cohort included 22,894 volunteers. To assure a minimum follow-up of 2 years and 9 months (to allow participants to fill the first follow-up questionnaire and account for the lag time in returning the questionnaire and avoid a potential selection bias), we only included participants recruited before March 2017. Out of 22,553 eligible participants, we excluded 450 individuals with an extreme total daily energy intake (<1st and >99th centiles) and 1,437 participants were lost to follow-up with a retention rate of 93%. For the present analysis, we included 20,666 participants (Figure 1).

### Covariate assessment

At baseline, volunteers completed a self-administrated questionnaire that includes information related to sociodemographic (marital status, years of university education, others), anthropometric measurements (weight and height), lifestyle variables (smoking habits, alcohol intake, physical activity, amount of time spent on screen devices, others), as well as family and personal medical history. NCDs such as diabetes, cancer, and cardiovascular events were confirmed by medical reports. Self-reported measurements and diagnosis at baseline and during the follow-up were previously validated in a subsample of the cohort and have been found reliable (18–21).

### Outcome ascertainment

The primary end-point was mortality from all causes, including CVD and cancer. Deaths were reported by next-of-kin, professional associations, or the postal system authority, which permitted us to identify more than 85% of deaths. For the rest of the deceased, the Spanish National Death Index was checked at least once a year to confirm the vital status of the participants and to request data about the cause of death. Trained physicians, blinded to the exposure, classified the cause of death considering the International Classification of Diseases (10th version) based on the data provided by the National Death Index.

### Dietary intake assessment

Dietary intake was self-reported by participants at baseline and after 10 years of follow-up through a validated 136-item food-frequency questionnaire (FFQ) (22, 23). The FFQ includes foods and beverages frequently consumed in Spain, such as dairy products and derivates, eggs, meat (fresh and processed), fish, seafood, vegetables, fruits, legumes and cereals, oils and fats, pastries, beverages (alcoholic, sugar-sweetened, and artificially sweetened beverages), and a miscellaneous group. Participants were asked to report on average over the past year their frequency of consumption, considering a specific typical Spanish serving per day (slice, teaspoons, glass, and others) for each of 136 food items. The nutrient composition of the dietary intake was assessed based on Spanish food composition tables (24, 25). Frequency of consumption was split into nine categories ranging from never/almost never to >6 servings/day for each FFQ item. Daily consumption was calculated by multiplying portion size by frequency. Nutrient intakes were computed as the sum of the frequency of consumption (converted to daily intake) of each item multiplied by the nutrient composition of specified portion size. Adherence to the Mediterranean Diet (MedDiet) was evaluated using the well-known 0–9 Mediterranean Diet Score (26, 27).

### Warning label score

**FIGURE 2:**
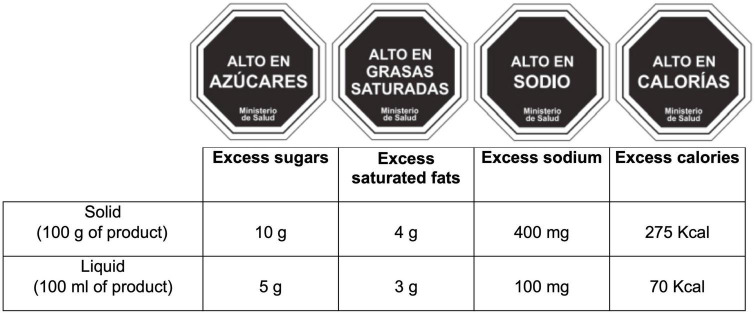
The four approved warning labels in Chile implemented as front-of-package labels and Chilean score computation for warning labels in pre-packaged food and beverage. All octagons indicate “Ministry of health”.

The FoP nutrition label was established by the Chilean Government, according to the regulation of pre-packaged foods in 2019 (Figure 2) as part of a unique law mandating warning labels, restricting marketing, and regulating school sales for products classified as nutritionally unhealthy. The black-and-white stop sign (octagons) labels use data from the nutritional declaration for 100 g or 100 ml of product and include the expression “High in” if the amount of added sugar, sodium, saturated fat, and/or calories exceeds the acceptable thresholds (Figure 2; 9, 28, 29), according to the Spanish food composition in the case of our study (24, 25). Products are required to carry a stop sign warning for each nutrient exceeded, meaning some products can require up to four labels. The warning label should be placed on any place of the package if the surface is between 30 and 60 cm^2^, and in the main container package if the product is smaller than 30 cm^2^. The food groups considered in the present study (shown in Supplementary Table 1) correspond to pre-packaged products, including processed and ultra-processed, as well as fats such as margarine, butter, and lard. Processed foods include canned or bottled foods (legumes, vegetables, and fruits) preserved in salt or syrup; canned sardine or tuna; salted, smoked, or cured meat or fish; cheeses; and bread and baked products. Ultra-processed products comprise carbonated soft, sweetened drinks, or juices; sweet, salty, or fatty packaged snacks; biscuits (cookies and cakes); ice cream; candies (confectionery); sweetened breakfast cereals; sugared milk; and products ready to eat (instant soups, noodles, desserts, sausages, burgers, pizza, and all pre-prepared meat) (29, 30). We considered the density of liquid products (milkshakes, sugar-sweetened and artificially sweetened beverages, and bottle juice) as 1 g/ml. Only added sugars were considered to evaluate excess sugar for dairy products, not considering lactose. The products of the FFQ that exceeded any of the nutrient thresholds per 100 g/ml (Figure 2) were assigned the respective warning label (Supplementary Table 1). For the calculation of the warning labels, the average of the critical nutrients based on the Spanish food composition tables contained in each food item of the FFQ was used (e.g., the critical nutrients of sausages were estimated as the average of nutrients from the consumption of sausages, chorizo, and mortadella). At the individual level, the total score of warning labels was computed as the sum of the warning label of each food or beverage consumed divided by 100 g/100 ml of food/liquids:


(1) $$\text{Warning label score (per day)}=\sum_{i=1}^{n}\left(\frac{{\text{WS}}_{i}I_{i}}{100}\right)$$

WS represents the number of warning labels of each food/beverage of the FFQ, *i* per 100 g/ml of product, and *I_i_* the total intake of each food/beverage in grams or millilitres per day.

### The Nutri-Score nutrient profile

The computation of the Nutri-Score FOP labelling has been described elsewhere (6, 31, 32). In brief, the algorithm allocated points based on the nutrient content of 100 g/ml of a food or beverage (6, 12, 13, 16, 33–35). For the content of critical nutrients, which are relevant for the risk of NCD, 0–40 points were allocated (0–10 points for each following components: sugars [g], saturated fats [g], sodium [mg], and energy [kJ]) and 0–15 points were allocated for the content of beneficial nutrients that should be promoted (0–5 points for each component: fibres [g], proteins [g], and the percentage of vegetables, fruits, legumes, nuts, rapeseed, walnut, and olive oils that compose the total product [%]) (6). The total food/beverage-level score was computed by subtracting the content of nutrients that should be consumed in limited amounts (negative points) from the nutrients that should be encouraged (positive points) (6). Therefore, the final FSAm-NPS score for each food/beverage range from −15 (most healthy) to +40 (least healthy). The individual-level score was calculated as the sum of the FSAm-NPS score for each food/beverage consumed multiplied by the quantity of energy supplied by that product divided by the sum of energy consumption from all foods (6).

### Statistical analyses

Participants were classified according to quartiles of consumption of the warning label score at baseline and for the repeated measurement analyses at 10 years of follow-up. Categorical and continuous variables are presented as percentages or means (standard deviation), respectively. Cox proportional hazard regression models with age as the underlying time variable (birth date as origin) were fitted to evaluate the potential association between quartiles of the warning label score and mortality, including all-cause, CVD, and cancer. Participants contributed person-time to the model from the date of returning the baseline questionnaire until the date of death, loss to follow-up, or when the last follow-up questionnaire was completed, whichever event occurred first. To minimize any measurement errors for variations in the dietary pattern during the follow-up, we performed time-dependent Cox regression models using repeated measures, considering cumulative average data and updated information on dietary consumption of the 10-year follow-up questionnaire for volunteers with follow-up longer than 10 years. The Pearson correlation coefficient between the score of warning labels at baseline vs. 10 years was moderate (*r* = 0.37; *p* < 0.001). For the analyses of CVD and cancer mortality, we excluded deaths attributable to other causes to rule out residual confounding. Hazard ratios (HR) and 95% CIs were estimated using the first quartile as the reference category. Multivariable models were stratified for deciles of age and recruitment period. After sex and age-adjusted analyses, multivariable models were also adjusted for marital status (married yes/no), physical activity evaluated as METs-h/week (continuous), alcohol intake (g/d, continuous), smoking status (never, current, and former), pack-years of cigarette smoking (continuous), snacking between meals (yes/no), following a special diet at baseline (yes/no), body mass index (linear and quadratic terms), total energy intake (quartiles), years of university education (continuous), family history of cardiovascular disease (CVD) and cancer, prevalent CVD, hypertension, diabetes, cancer and depression, and self-reported hypercholesterolemia at baseline. For the variable smoking pack-years, 7.9% of data were missing, and we applied simple imputations using as predictor variables age, sex, physical activity, years of university, BMI, alcohol consumption, adherence to the MedDiet, and mortality. A Linear trend test was conducted across quartiles assigning the median value to each category and treating them as a continuous variable. Furthermore, stratified analyses were carried out according to sex (men or women); age at recruitment (<45 or ≥45 years); baseline BMI (<25 or ≥25 kg/m^2^); and smoking habits at baseline (ever or never smoker). To evaluate the robustness of our findings, we applied the following sensitivity analyses: considering different plausible energy limits proposed by Willett (36), as well as percentiles 5–95 to avoid information bias due to over or under-reporters; excluding volunteers who had prevalent conditions at baseline (CVD, cancer, and diabetes); omitting premature death (if it occurred earlier than 2-year follow-up); excluding snacking between meals as a confounder; and additionally adjusted for fibre intake. Restricted cubic splines with three knots, considering zero as reference were applied to the flexible model to graphically represent the dose-response association between mortality and the warning label score (as continuous), as well as to evaluate non-linearity.

Exploratory analyses were performed to assess the effect of different food policy approaches on the risk of all-cause mortality. For this purpose, participants were categorized under or above the median (≥*p*50*^th^*) of the warning label score or Nutri-Score, and >4 servings/day of UPF based on previously published studies from our cohort (5, 6). We also evaluated multiple interactions between them by testing an interaction product term with the maximum likelihood ratio test. To find out the ability of predicting the relationship between all-cause mortality and the nutrient profiles of the warning label score and Nutri-Score, we categorized participants into quartiles of both FoP labels and compared the fourth vs. the first quartile of these exposures using the Akaike Information Criteria (AIC) and Bayesian Information Criteria (BIC) in the adjusted model previously mentioned. All tests were two-sided and statistical significance was set at the conventional 0.05 level. Analyses were performed using STATA version 16.0 (StataCorp, College Station, TX, USA).

## Results

A total of 20,666 participants, including 8,068 men and 12,598 women (Figure 1) with an average of 38 [12.4] years (mean [SD] age at baseline), were analysed. After a median follow-up of 12.2 years (238,217 person-years), a total of 467 overall deaths were registered of which 90 (19.3%) were attributed to CVD, 242 (51.8%) to cancer, 137 (26.1%) to other causes, and 13 (2.8%) to unknown causes. Table 1 shows baseline characteristics of the cohort study categorized by quartiles of warning label score. Participants in the highest quartile were more likely to be men, never smokers, spend more time watching TV and using computer, snack between meals, and have a lower prevalence of diabetes, cancer, and CVD at baseline compared to the lowest quartile. Regarding dietary components, individuals with a higher warning label score in their diet had an increased energy intake, saturated fats, sodium, and alcohol, as well as lower adherence to the MedDiet than those in the lowest category. Moreover, these participants (Q4) had a slightly low intake of vegetables, fruits, and low-fat dairy products compared to those in Q1. Meanwhile, individuals in the fourth quartile of the warning label score (Q4) showed slightly higher fibre consumption than participants in the first quartile. This result could be attributed to the fact that the foods included in the warning label score are not related to sources rich in fibre, such as fruits, vegetables, dry fruits, and others. On average, participants in the highest quartile had a higher intake of white bread, dairy products, red and processed meat, and UPF foods than individuals in the lowest quartile.

**TABLE 1:** Baseline characteristics of participants according to quartiles of the score calculated for Chilean warning labels: the SUN (Seguimiento Universidad de Navarra) cohort.

Variable	Quartiles of the score for Chilean warning labels			
	
	Q1	Q2	Q3	Q4
N	5167	5167	5166	5166
Age, years	41.6 (13.0)	38.6 (12.3)	36.7 (11.6)	35.6 (11.7)
Sex, men (%)	35.1	35.5	39.2	46.4
Score of Warning labels (per day)	3.6 (1.0)	6.0 (0.6)	8.3 (0.8)	13.2 (3.4)
BMI (kg/m^2^)	23.8 (3.6)	23.5 (3.5)	23.3 (3.5)	23.4 (3.6)
Married (%)	55.2	52.5	49.2	43.0
Years of university education	5.0 (1.5)	5.1 (1.5)	5.0 (1.5)	5.0 (1.5)
**Smoking status, n (%)**				
Never	44.2	48.2	50.7	52.5
Current	20.3	21.0	22.4	24.1
Former	35.5	30.8	26.9	23.4
Physical activity (METs-h/week)	22.1 (23.5)	21.5 (21.4)	21.8 (23.0)	22.4 (24.6)
**Screen time**				
Television viewing (h/day)	1.6 (1.1)	1.6 (1.1)	1.6 (1.2)	1.7 (1.2)
Computer time (h/day)	2.0 (1.9)	2.1 (1.9)	2.1 (1.9)	2.2 (2.0)
**Conditions at baseline**				
Diabetes (%)	3.2	1.6	1.3	1.0
Cancer (%)	3.5	2.4	2.3	1.8
Hypertension (%)	14.6	11.1	9.3	9.3
Cardiovascular disease (%)	2.4	1.6	1.1	1.2
Hypercholesterolemia (%)	22.1	16.9	15.3	13.8
Depression (%)	13.5	11.2	10.8	11.0
Family history of CVD (%)	16.2	13.8	12.8	12.4
Family history of cancer (%)	17.3	15.6	14.2	13.9
Following special diets (%)	15.3	7.6	5.1	4.3
Between-meal snacking (%)	25.2	31.5	37.2	43.5
**Dietary nutrient profile**				
⍭Adherence to MedDiet (0–9 points)	5.1 (1.7)	4.7 (1.7)	4.4 (1.7)	4.2 (1.6)
Total energy intake (kcal/day)	1824 (481)	2231 (459)	2618 (493)	3343 (709)
Carbohydrate (%)	42.3 (8.5)	43.0 (7.1)	43.5 (6.7)	44.8 (7.1)
Protein (%)	19.8 (3.8)	18.4 (2.9)	17.5 (2.6)	16.4 (2.7)
Fat (%)	35.4 (7.4)	36.5 (6.3)	37.1 (6.0)	37.2 (6.2)
Monounsaturated fatty acids (g/day)	31.9 (12.9)	39.1 (12.5)	46.0 (13.6)	57.8 (18.0)
Polyunsaturated fatty acids (g/day)	22.5 (8.0)	30.1 (8.1)	37.0 (9.6)	49.6 (17.1)
Saturated fatty acids (g/day)	9.9 (4.5)	12.6 (4.8)	15.6 (5.7)	20.5 (7.9)
Total dietary fibre intake (g/day)	27.4 (14.3)	28.2 (13.4)	29.6 (13.6)	33.3 (14.5)
Sodium intake (mg/day)	2552 (1016)	3453 (1253)	4321 (1697)	6368 (4023)
Alcohol intake (g/day)	6.3 (10.4)	6.5 (9.7)	6.8 (10.2)	7.6 (11.9)
**Food consumption**				
Vegetables (g/day)	567 (389)	547 (359)	540 (364)	549 (381)
Fruits (g/day)	377 (351)	364 (312)	362 (340)	368 (343)
Total nuts (g/day)	7.8 (17.5)	7.5 (13.2)	8.4 (14.6)	9.1 (13.1)
Vegetable fat (g/day)	18.5 (15.2)	20.3 (15.9)	21.9 (16.7)	24.1 (18.6)
Olive oil (g/day)	17.4 (14.7)	18.8 (15.1)	19.9 (15.5)	21.5 (17.3)
^‡^Animal fat (g/day)	1.9 (4.0)	3.1 (5.3)	4.1 (7.3)	5.8 (13.1)
Legumes (g/day)	22.3 (20.6)	22.4 (17.3)	24.2 (20.4)	25.7 (20.8)
White bread (g/day)	25.2 (29.8)	49.8 (46.6)	70.2 (59.5)	109.2 (99.8)
Whole grain bread (g/day)	14.5 (29.2)	15.0 (33.4)	13.1 (34.0)	14.0 (39.9)
Dairy products (g/day)	125 (165)	178 (188)	228 (205)	301 (251)
Low-fat dairy products (g/day)	252 (258)	239 (252)	225 (244)	216 (264)
Fish and shellfish (g/day)	102 (67.4)	101 (74.9)	101 (68.4)	103 (71.8)
Red meat (g/day)	64.1 (47.6)	75.1 (44.0)	84.9 (49.5)	95.5 (55.5)
Processed meat (g/day)	28.8 (20.9)	40.6 (26.0)	50.2 (29.7)	66.1 (49.4)
Ultra-processed food (g/day)	12.9 (14.2)	20.1 (17.7)	25.2 (22.1)	33.0 (33.3)
Pastries (g/day)	24.0 (20.7)	41.5 (29.2)	58.6 (38.6)	98.1 (79.9)
Sugar sweetened beverages (g/day)	21.4 (36.6)	43.4 (57.7)	68.1 (82.3)	137.1 (191.0)
Nutri-Score	2.8 (1.8)	4.2 (1.6)	5.1 (1.5)	6.2 (1.8)

MET, metabolic equivalent of task.

⍭9-item Mediterranean Diet Score proposed by Trichopoulou et al. (27, 28).

^‡^Sum of butter, lard, and cream.

**FIGURE 3:**
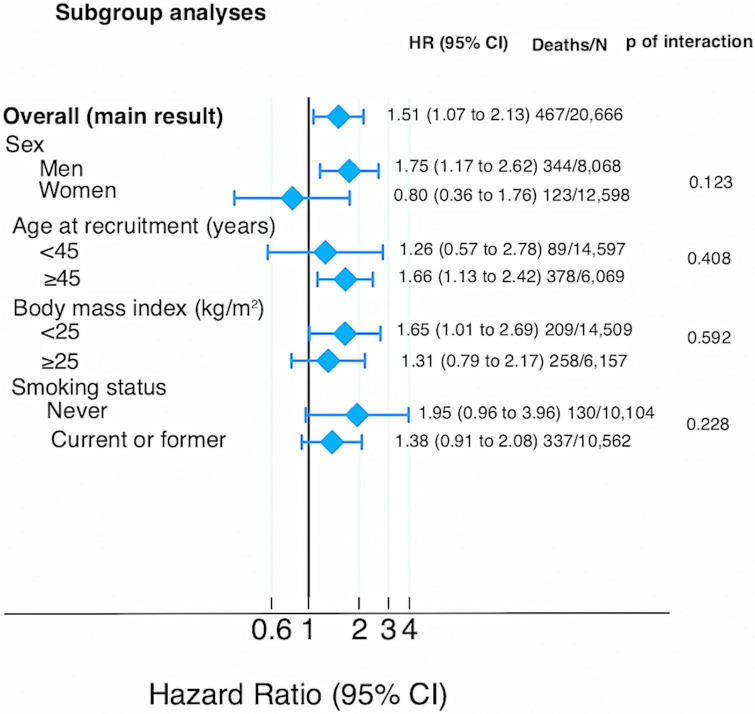
Sub-group analyses for the association between Chilean warning label score and all-cause mortality (multivariable-adjusted HR and 95% CI for the highest vs. lowest quartile).

**FIGURE 4:**
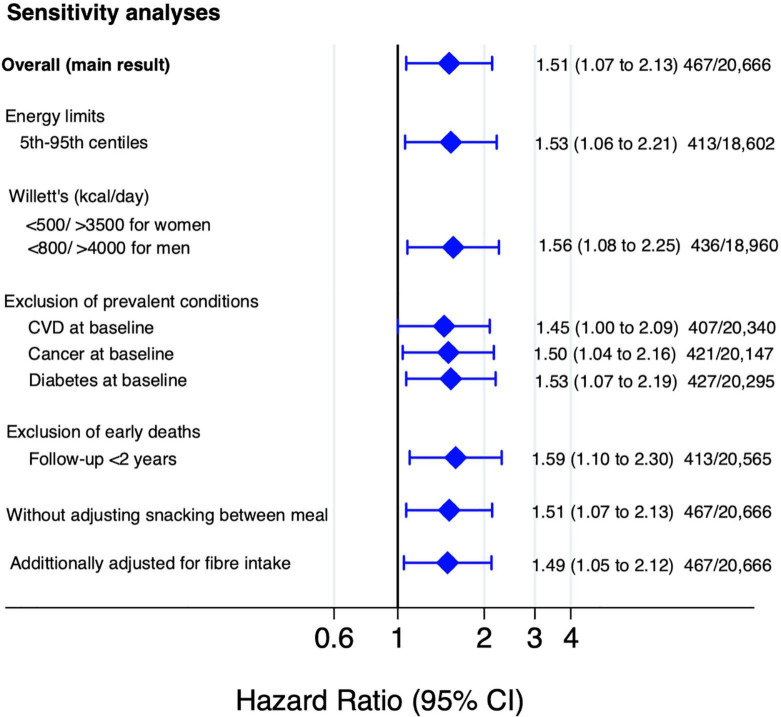
Sensitivity analyses for the association between Chilean warning label score and all-cause mortality (multivariable-adjusted HR and 95% CI for the highest vs. lowest quartile).

**FIGURE 5:**
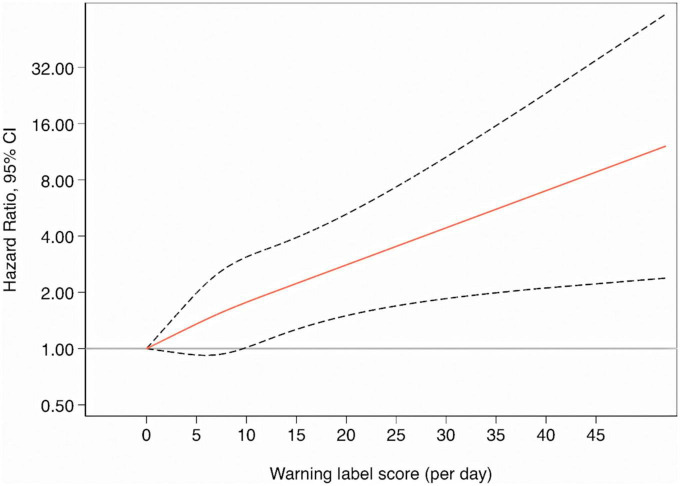
The smooth line represents the hazard ratio for the risk of all-cause mortality when using zero as the reference value for the warning label score (3 knots) whereas the dashed lines indicate 95% CIs.

**FIGURE 6:**
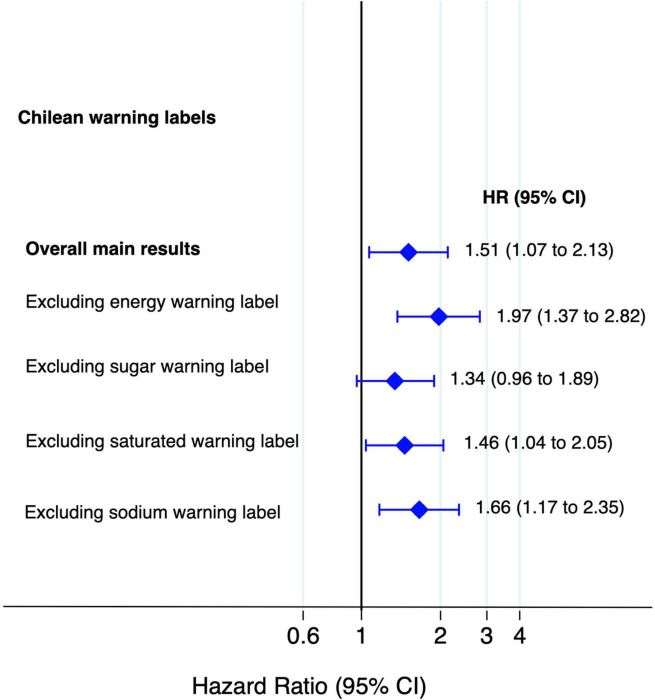
Association between food consumption with Chilean warning label score and mortality and excluding one warning label at time (multivariable-adjusted HR and 95% CI for the highest vs. lowest quartile).

The HRs for all cause and cause specific mortality according to quartiles of the score of Chilean warning labels are presented in Table 2. Higher score of these warning labels (Q4) was associated with an increased risk of all-cause mortality compared to the lowest quartile (Q1: reference) in the fully adjusted model: 1.51 (95% CI: 1.07–2.13), and there was a linear dose-response relationship across quartiles (*p*-trend = 0.010). Moreover, the multivariable-adjusted model showed that participants in the fourth quartile of warning labels had an increased risk of cancer mortality [HR: 1.91 (95% CI: 1.18–3.10; *p*-trend = 0.006)] compared to the first quartile. However, there was no statistically significant association between this score of warning label and CVD mortality [(HR: 1.20; 95% CI: 0.54–2.69; *p*-trend = 0.670)]. Repeated measurements, using data from food consumption after 10 years of follow-up evidenced that the highest quartile of warning labels was consistently associated with a significantly higher risk to all-cause [(HR: 1.47; 95% CI: 1.04–2.08; *p*-trend = 0.089)] and cancer mortality [(HR: 1.84; 95% CI: 1.14–2.96; *p*-trend = 0.028)] as compared to the lowest quartile. Figure 3 shows the relationship between food consumption with the warning label score and overall mortality in subgroup analyse comparing the fourth vs. the first quartile. However, we did not find statistically significant interactions (all *p*-values > 0.1). To test the robustness of our main findings, several sensitivity analyses were conducted (Figure 4) after considering different scenarios. Results did not change in any of the different scenarios, suggesting that the association between higher scores of the Chilean warning label nutrient profile and all-cause mortality was robust. Nonetheless, when we excluded cases of prevalent conditions (CVD, cancer, and diabetes), the associations were no longer significant. Figure 5 shows the dose-response relationship between intake of warning labels and all-cause mortality. The restricted cubic spline model indicated that individuals who had more than 10 warning labels had a higher risk of all-cause mortality (Figure 5). In addition, we evaluated the relative influence of each of the warning labels by repeating the multivariable-adjusted Cox regression models excluding one warning label at a time and comparing the highest vs. lowest quartile (Figure 6). All HRs showed a direct association between mortality and higher intake of warning labels, but interestingly no significant association was found when excluding sugar warning labels (HR: 1.34; 95% CI: 0.96–1.89). Analyses combining exposures (warning label, UPF, or Nutri-Score) are shown in Table 3. Participants in the highest categories (warning label ≥ *p*50th and UPF > 4 servings/day) presented a 66% increased risk of mortality [HR (95% CI): 1.66 (1.21–2.26)] compared to the lowest category. Similar results were found for the highest score of warning label and Nutri-Score (both ≥ *p*50th) [HR (95% CI): 1.51 (1.14–2.01)], as well as when we evaluated the Nutri-Score and UPF intake [HR (95% CI): 1.60 (1.22–2.11)] (Supplementary Table 2). When comparing the warning label score and Nutri-Score (Table 4), fully adjusted models showed that the AIC and BIC values did not differ from each other when we compared the highest vs. lowest quartile. For the warning label: AIC = 4264, BIC = 4470; and for the Nutri-Score: AIC = 4266, BIC = 4472.

**TABLE 2:** Cox proportional hazard ratios (95% confidence intervals) for mortality according to quartiles of Chilean warning label score.

	Q1	Q2	Q3	Q4	*P* for trend
Daily warning label score	0–5.0	>5.0–7.1	>7.1–9.8	>9.8	
**All-cause mortality**					
N° participants	5,167	5,167	5,166	5,166	
Person-years	57,414	59,099	60,743	60,961	
N° deaths	156	98	100	113	
Age and sex adjusted	1.00 (Ref)	0.83 (0.65–1.06)	1.01 (0.78–1.30)	1.07 (0.83–1.37)	0.391
Multivariable adjusted	1.00 (Ref)	0.97 (0.74–1.27)	1.32 (0.95–1.83)	1.51 (1.07–2.13)	0.010
Repeated measurements of diet	1.00 (Ref)	1.02 (0.78–1.32)	1.32 (0.96–1.81)	1.47 (1.04–2.08)	0.089
**CVD mortality**					
N° participants	5,049	5,090	5,078	5,072	
Person-years	56,390	58,461	59,975	60,098	
N° deaths	38	21	12	19	
Age and sex adjusted	1.00 (Ref)	0.73 (0.42–1.25)	0.53 (0.28–1.01)	0.79 (0.44–1.42)	0.348
Multivariable adjusted	1.00 (Ref)	0.87 (0.49–1.54)	0.77 (0.34–1.75)	1.20 (0.54–2.69)	0.670
Repeated measurements of diet	1.00 (Ref)	0.99 (0.57–1.73)	0.74 (0.32–1.72)	1.16 (0.51–2.63)	0.930
**Cancer mortality**					
N° participants	5,084	5,123	5,123	5,111	
Person-years	56,697	58,706	60,425	60,474	
N° deaths	73	54	57	58	
Age and sex adjusted	1.00 (Ref)	0.97 (0.68–1.39)	1.19 (0.84–1.68)	1.27 (0.89–1.81)	0.122
Multivariable adjusted	1.00 (Ref)	1.14 (0.78–1.68)	1.50 (0.96–2.35)	1.91 (1.18–3.10)	0.006
Repeated measurements of diet	1.00 (Ref)	1.16 (0.79–1.71)	1.48 (0.95–2.28)	1.84 (1.14–2.96)	0.028

Ref, reference. Multivariate model adjusted for age (underlying time variable), sex, marital status (married), physical activity (continuous), alcohol intake (g/d, continuous) smoking status (never, current, and former), pack-years of cigarette smoking (continuous), snacking (dichotomous), special diet at baseline (dichotomous), body mass index (linear and quadratic terms), total energy intake (quartiles), years of university education (continuous), family history of cardiovascular disease (CVD) and cancer, prevalent CVD, hypertension, diabetes, cancer and depression, self-reported hypercholesterolemia at baseline. Stratified by deciles of age and recruitment period. Multivariable adjusted with repeated measures were adjusted for the same variables with updated data at 10 years of follow-up (smoking, energy, and alcohol intake).

**TABLE 3:** Hazard ratios (95% confidence intervals) for all-cause mortality according to baseline median consumption of foods with Chilean warning labels, ultra-processed food (UPF), and Nutri-Score.

Score of the Chilean warning label	UPF consumption			Nutri-Score		
			
	(≤4 servings/day)	(>4 servings/day)	*p for interaction	<*p*50th (<4.6)	≥*p*50th (≥4.6)	*^¶^ p* for interaction
**<*p*50th (<7.1)**						
N of deaths/N of participants	242/9,727	14/649	0.738	200/7,576	56/2,800	0.076
Multivariable	1.00 (Ref)	1.46 (0.80–2.67)		1.00 (Ref)	0.95 (0.71–1.29)	
**≥*p*50th (≥7.1)**						
N of deaths/N of participants	97/3,819	114/6,471		63/2,767	148/7,523	
Multivariable	1.27 (0.96–1.69)	1.66 (1.21–2.26)		1.08 (0.77–1.50)	1.51 (1.14–2.01)	

Multivariate model adjusted for age (underlying time variable), sex, marital status (married), physical activity (continuous), alcohol intake (g/d, continuous), smoking status (never, current, and former), pack-years of cigarette smoking (continuous), snacking (dichotomous), special diet at baseline (dichotomous), body mass index (linear and quadratic terms), total energy intake (quartiles), years of university education (continuous), family history of cardiovascular disease (CVD) and cancer, prevalent CVD, hypertension, diabetes, cancer and depression, and self-reported hypercholesterolemia at baseline. Stratified by deciles of age and recruitment period. *p for interaction: UPF; ^¶^ *p* for interaction: Nutri-Score.

**TABLE 4:** Comparison of AIC and BIC values for the warning label score and Nutri-Score in relation to all-cause mortality.

	Criterion		
	
	HR (95% CI)	AIC	BIC
**Chilean warning label**			
Q4 vs. Q1	1.51 (1.07–2.13)	4264	4470
**Nutri-Score**			
Q4 vs. Q1	1.40 (1.05–1.87)	4266	4472

HR, hazard ratio; AIC, Akaike’s information criteria; BIC, Bayesian information criteria.

## Discussion

The results of the present study provide new insights into the association between the nutrient profile underpinning the warning label score and mortality in a large prospective Spanish cohort. To our knowledge, this is the first study that suggests that higher consumption of foods with warning labels (>9.8/day) (poorer nutritional quality), indicating a lower healthiness, is significantly associated with the risk of all-cause and cancer mortality. These findings add to previous studies evaluating the relationship between the Nutri-Score FoP label and health outcomes in other prospective cohorts, including the SUN project. Gómez-Donoso et al. showed that higher FSAm-NPS (nutritional algorithm underpinning the Nutri-Score) characterised by a lower diet quality was associated with a higher risk of all-cause mortality [HR highest vs. lowest quartile (95% CI): 1.82 (1.34–2.47); *p*-trend < 0.001] and cancer mortality [HR highest vs. lowest quartile (95% CI): 2.44 (1.54–3.85); *p*-trend < 0.001] in the SUN cohort (6). Moreover, some studies of the European Prospective Investigation into Cancer and Nutrition (EPIC) cohort study have shown an association between a higher FSAm-NPS intake and a higher risk of cancer and higher rates of mortality overall and from cancer and diseases of the circulatory, respiratory, and digestive systems (12, 13).

A modelling study predicted the effect of warning labels on obesity reduction in Mexico 5 years after implementation (37). Investigators estimated a caloric reduction of an average of 36.8 kcal/person/day from beverages and snacks, which could reduce obesity rates by 14.7% (37). These results are relevant considering that obesity is recognized as a major risk factor for several cancers, CVDs, and premature death (38, 39). Our findings did not show a significant association between the nutrient profile of warning labels and CVD mortality, one possible explanation for this finding may be the lack of statistical power to detect any significant association in CVD mortality due to the low number of cases of CVD deaths. In addition, in the sensitivity analysis excluding participants with CVD at baseline, the statistical significance between warning label score and total mortality was weaker probably because the number of total deaths decreased. However, an increased intake of UPF was related to a higher warning label score. This result might be explained by the fact that the nutrient profile of the warning labels takes into account critical nutrients and energy content, a nutritional composition dimension closely related to the UPF. Bonaccio et al. reported that higher intake of UPF was directly associated with CVD mortality (highest vs. lowest quartile: 1.58; 95% CI: 1.23–2.03) and death from ischemic heart disease/cerebrovascular disease (highest vs. lowest quartile: 1.52; 95% CI: 1.10–2.09) among participants from Italy with a mean age of 55 years (40). It has to be mentioned that UPF are industrially manufactured ready-to-eat or heat foods that usually use industrial processes, modification of the food matrix, and food additives, leading to the production of several components closely related to CVD (41). Previous studies in the SUN cohort showed that the highest intake of UPF was associated with an increased risk of all-cause mortality (5) (HR: 1.62; 95% CI: 1.13–2.33) and obesity (42) (HR: 1.26; 95% CI: 1.10, 1.45) compared to the lowest intake of UPF. Also, our analyses showed that participants who had increased intake of UPF (≥4 servings/d) and warning label score (≥5.5) presented an HR of 1.66 (95% CI: 1.21–2.26) for overall mortality. Possible mechanisms are hypothesized to include disrupted renal sodium homeostasis, metabolic and hemodynamic modifications, alteration of the gut microbiota, glycaemic response and insulin sensitivity, and so on (41). Regulatory policies are needed to mitigate the impact of UPF on NCDs, depression, all-cause mortality, and other diseases (43).

The existing scientific evidence has consistently shown that foods with low diet quality, as estimated by FoP nutrition labelling systems, are closely related to the increased risk of NCDs and deaths (6, 12–15, 44). In this sense, it is noteworthy to mention that the warning label score and Nutri-Score have AIC and BIC values close to each other, indicating that the nutrient profiles of these FoP systems have similar power to predict mortality in our sample. Caution is needed in interpreting this result, considering that the scores at the individual level could vary across populations (e.g., different dietary patterns), but our results at least suggest that the nutrient profile and thresholds of warning label and Nutri-Score could highlight less healthy food products. In addition, individuals with warning labels and Nutri-Score values above median (combined exposures) exhibited a 51% increased risk of all-cause mortality. These FoP labels differ in the type of information provided (nutrient-specific or summary) and label format, and both are calculated per 100 g/ml of product (7). The Nutri-Score is a colour-coded graded scale ranging from higher (dark green) to lower nutritional (dark orange) quality based on five letters (from A to E) (7). Meanwhile, the warning systems focus on excessive amounts of “critical nutrients” (7, 10). The Nutri-Score is the FoP system most used in Europe. Egnell et al. evaluated five nutrition labelling systems across 12 countries and showed that the Nutri-Score has better performance to rank products considering their nutritional quality followed by the Multiple Traffic Lights, Health Star Rating system, Warning symbol, and the Reference Intakes (45). However, other studies indicated differences in consumers’ understanding across diverse FoP nutrition label schemes (29, 45–47). It is also important to mention that consumers’ preferences and perceptions could vary among countries, depending on cultural behaviour (11). Previous studies have reported that consumers with the highest income, education levels, and nutrition knowledge tend to have higher levels of awareness, which influenced their capability to use FoP nutrition labels (11). On the other hand, it is increasingly recognized that the warning label system is related to a decrease in purchases of packed products higher in calories and nutrients of concern (10, 46, 48, 49). These FoP warning labels have been increasingly adopted in Latin American countries such as Chile, Ecuador, Mexico, Peru, and Uruguay (7, 10). Studies conducted in Chile show that there has been a decrease in products that need the warning labels “high in sodium” and “high in sugar,” suggesting the tendency of food reformulation by the food industry (50).

Non-communicable diseases have rapidly risen around the world with a negative impact on burden diseases and premature deaths, leading to disproportions in low- and middle-income countries, which constitutes a public health challenge (51). The global tendency exhibits an increased volume and market of fast food and highly processed products in parallel with obesity rates (52). The FoP nutrition labels are part of a set of recommended policies aimed at reducing the global burden of diet-related NCDs by promoting market regulation, innovation-reformulation of packaged products, and fiscal measures (7, 8, 10, 52–54). There is no doubt that the implementation of FoP labelling has consistently proven to improve the ability to identify the healthfulness of food choices compared to no label (7, 8), suggesting that FoP labels play a pivotal role in improving the healthiness of food purchases and contribute to improving population diets.

The strengths of our study are the prospective and dynamic design, as well as the long follow-up and good retention rate (93%) of many participants from a Mediterranean country. Furthermore, the SUN cohort collects a wide range of potential confounders (sociodemographic and lifestyle data), and the analyses include the use of repeated measures of diet and the performance of exhaustive sensitivity analyses. Nevertheless, the present study has some limitations. First, the SUN cohort encompasses Spanish graduates who have high education levels. Thus, our sample is not representative of the general population, but this is an advantage in that the homogeneous university graduate cohort decreases the likelihood of misclassification bias. Second, the self-reported FFQ used for dietary data could be susceptible to misclassification. However, this FFQ has been previously validated in independent cohorts (22, 23, 55). Third, we could not evaluate specific food products that participants consumed (brand of the product and their variability in the nutrient content of the products inside each food item) or some features of manufactured products (e.g., level of processing) and unpackaged foods (e.g., homemade recipes rich in critical nutrients). Thus, it could have resulted in some misclassification of our exposures. Nonetheless, the FFQ used covered the main food groups of the usual dietary consumption of participants. Additionally, we used Spanish food composition tables, which enclosed representative values of main foods products consumed by the Spanish population. Last, although we included many potential confounders, the observational design can never completely rule out residual confounding bias.

## Conclusion

A diet including foods with a higher score of warning labels (indicating a lower nutritional quality) was a good predictor of all-cause and cancer mortality in Spanish population. Also, the nutrient profiles of the warning label score and Nutri-Score have a similar power of prediction. Therefore, our results reinforce the suitability of FoP warning labels as a key policy action to improve health status and prevent NCDs.

## Future directions

As a policy response to prevent NCDs, governments should implement FoP nutrition labelling to enable consumers to make healthier food choices and encourage the food industry to reformulate products to be healthier. The FoP warning label is used in many countries (7, 10), showing positive effects on consumer’s choice, and from these results also finding a direct effect with better health. However, future research should be aimed at evaluating the consumption of foods with higher warning labels and NCDs in other ethnicities.

## Data availability statement

The raw data supporting the conclusions of this article will be made available by the authors, without undue reservation.

## Author contributions

VB-V, MB-R, CS-O, and MAM-G: conceptualization, methodology, and formal analysis. VB-V: writing—original data. All authors interpretation of data, writing—review and editing, read, and agreed to the published version of the manuscript.
