# Supplementary material for: Mortality prediction of the nutrient profile of the Chilean front-of-pack warning labels: Results from the Seguimiento Universidad de Navarra prospective cohort study
Source: Front Nutr. 2022 Oct 21;9:951738. doi: 10.3389/fnut.2022.951738 (PMC9633686; doi:10.3389/fnut.2022.951738)
Supplement: Supplementary file 1 [file Table_1.pdf]

Supplementary Material

Table S1. Warning label score from FFQ items from the SUN cohort for each 100g of product

	Chilean warning labels				
**Food/beverage item**	**Excess calories**	**Excess sugars**	**Excess saturated fats**	**Excess sodium**	**Total score**
Cream cheese	1	0	1	1	3
Condensed milk	1	1	1	0	3
Custard, flan and pudding	0	1	0	0	1
Fresh cheese	0	0	1	1	2
Curd	0	0	0	0	0
Ice cream	0	1	1	0	2
Petit suisse	0	0	0	0	0
Milkshake	1	0	0	0	1
Yogurt	0	0	0	0	0
Cream	1	0	1	0	2
Cured and semi-cured cheese	1	0	1	1	3
Cooked ham	0	0	0	1	1
Salted or smoked fish	0	0	0	1	1
Foie gras	1	0	1	1	3
*Sausages	1	0	1	1	3
Hamburger	0	0	1	1	2
Sobrasada	1	0	1	1	3
Serrano ham	0	0	0	1	1
Bacon	1	0	1	1	3
Blood puding	1	0	1	1	3
Sausages- Frankfurt	1	0	1	1	3
French fries	1	0	1	1	3
Fruits in syrup	0	1	0	0	1

Fuits in juicy	0	0	0	0	0
Breakfast cereal	1	1	0	1	3
Pizza	0	0	1	1	2
White bread	1	0	0	1	2
Whole bread	0	0	0	1	1
Lard	1	0	1	0	2
Margarine	1	0	1	1	3
Butter	1	0	1	0	2
Cookie	1	1	0	1	3
Marzipan	1	1	0	0	2
Other non-handmade confectionery	1	1	1	0	3
Muffin	1	1	1	0	3
Chocolate	1	1	1	0	3
Nougat	1	1	0	0	2
Croissants and others	1	0	1	0	2
Churros	1	0	0	0	1
Chocolate cookie	1	1	1	0	3
Doughnuts	1	1	1	0	3
Sugar-sweetened beverages	0	1	0	0	1
Bottled juice	0	1	0	0	1
Artificially-sweetened beverages	0	0	0	0	0
Fried foods	0	0	0	1	1
Tomato sauce	0	0	0	0	0
Mayonnaise	1	0	1	1	3
Jam	1	1	0	0	2
Hot sauce	0	0	0	1	1
Ready to eat soups	1	0	0	1	2

*Includes chorizo, and mortadella. For dairy products, the excess of sugar was assessed considering the content of free sugars.

Table S2. Hazard ratios (95% confidence intervals) for mortality according to baseline median of Nutri-Score and ultra-processed food (UPF) consumption

**Nutri-Score**	**UPF consumption**		
	(≤4 servings/day)	(>4 servings/day)	*p* for interaction
**<p50th** (<4.6)			
N of deaths/ N of participants	245/ 9,022	18/1,321	0.030
Multivariable	1.00 (Ref)	0.85 (0.48 to 1.49)	
**≥p50th** (≥4.6)			
N of deaths/ N of participants	94/4,524	110/5,799	
Multivariable	1.00 (0.78 to 1.29)	1.60 (1.22 to 2.11)	

Multivariate model adjusted for age (underlying time variable), sex, marital status (married), physical activity (continuous), alcohol intake (g/d, continuous) smoking status (never, current, and former), pack-years of cigarette smoking (continuous), snacking(dichotomous), special diet at baseline (dichotomous), body mass index (linear and quadratic terms), total energy intake (quartiles), years of university education(continuous), family history of cardiovascular disease (CVD) and cancer, prevalent CVD, hypertension, diabetes, cancer and depression, self-reported hypercholesterolemia at baseline. Stratified by deciles of age and recruitment period.
